# Purpura and Scurvy

**Published:** 1900-10-20

**Authors:** 


### PURPURA AND SCURVY.

In lecturing at Guy's Hospital a little time ago upon a case of purpura Dr. Hale White drew attention to the distinction between that disease and scurvy, a malady with which it was not infrequently confounded. Bleeding from the mouth, or from the gums, occurred in purpura; the test of scurvy was, he said, the peculiar sponginess of the gums. In this disease the gums and especially the lower ones swell up, so that they hide the teeth. On several occasions he had seen the gums, in cases of scurvy, so swollen that in order to make sure that they were there one had to pass the finger along the upper line and feel the teeth in the depression of the swollen gum. If the patient on whom he was lecturing had had scurvy, and had been as bad as he evidently was, it was very improbable that the sponginess of the gums would not have been well marked, and this point alone was enough to put scurvy out of court.

